# Retraction for Piracha et al., “Sirtuin 2 Isoform 1 Enhances Hepatitis B Virus RNA Transcription and DNA Synthesis through the AKT/GSK-3β/β-Catenin Signaling Pathway”

**DOI:** 10.1128/jvi.01186-25

**Published:** 2025-09-12

**Authors:** Zahra Zahid Piracha, Hyeonjoong Kwon, Umar Saeed, Jumi Kim, Jaesung Jung, Yong-Joon Chwae, Sun Park, Ho-Joon Shin, Kyongmin Kim

## RETRACTION

Volume 92, no. 21, e00955-18, 2018, https://doi.org/10.1128/jvi.00955-18. The authors hereby retract this article. Following a recent request to provide the original data underlying this publication, it was identified that several immunoblot images were reused within the article. This issue was not recognized at the time of submission and publication.

As the experiments were conducted more than 7 years ago, it is not possible to recover or verify the original experimental records.

Because of these unresolved concerns, we are retracting the article and regret any inconvenience caused to the readers.

